# A computed tomography algorithm for crossing coronary chronic total occlusions: riding on the wave of the proximal cap and distal vessel segment

**DOI:** 10.1007/s12471-020-01510-1

**Published:** 2020-11-11

**Authors:** M. P. Opolski, A. Nap, P. Knaapen

**Affiliations:** 1 Department of Interventional Cardiology and Angiology, National Institute of Cardiology, Warsaw, Poland; 2 Department of Cardiology, Heart Center, Amsterdam UMC, University of Amsterdam, Amsterdam Cardiovascular Sciences, Amsterdam, The Netherlands

**Keywords:** Coronary chronic total occlusion, Coronary computed tomography angiography, Percutaneous coronary intervention

## Abstract

With wider adoption of coronary computed tomography angiography (coronary CTA), chronic total occlusions (CTOs) are being increasingly identified and characterised by non-invasive angiography. In particular, the ability of coronary CTA to clearly delineate atherosclerotic plaque, as well as to display three-dimensional vessel trajectories, has garnered particular attention in the context of preprocedural planning and periprocedural guidance of CTO percutaneous coronary intervention (PCI). Single CTO features and combined scoring systems derived from CTA (mostly exceeding the diagnostic performance of the angiographic J‑CTO score) have been used to predict time-efficient guidewire crossing, and thus grade the CTO difficulty level prior to PCI. In addition, the introduction of three-dimensional CTA/fluoroscopy co-registration for periprocedural navigation during CTO PCI offers the unprecedented opportunity to resolve proximal cap ambiguity and clearly visualise the distal CTO segment, thereby potentially influencing CTO PCI strategies and techniques. In this review, the potential advantages of non-invasive evaluation of CTO by coronary CTA are described, and a CTA-based hybrid algorithm is introduced for further enhancing the efficiency of CTO PCI. Further studies are clearly needed to verify the proposed approach. However, several luminary operators have already implemented coronary CTA for planning and periprocedural guidance of CTO interventions using the hybrid algorithm.

## Introduction

A chronic total occlusion (CTO) may be identified either in patients with suspected coronary artery disease (CAD) undergoing coronary computed tomography angiography (coronary CTA) [1], or CTA may be specifically performed for a better characterisation of CTO previously detected on invasive angiography (usually for planning percutaneous or surgical interventions) [2, 3]. The prevalence of CTO detected on non-invasive CTA among patients with a low-to-intermediate probability of CAD is low but significant (reaching up to 6.2% among subjects with obstructive CAD), and increases relative to male sex, typicality of symptoms and CAD risk factors [1]. Whereas most individuals with CTO are managed medically, the presence of CTO on coronary CTA confers a worse prognosis with an increased rate of late revascularisation (but similar mortality) as compared with moderate-to-severe CAD [4].

Consistent with the angiographic definition of CTO, coronary CTA typically shows a lack of contrast opacification within the occlusion site with reversal of contrast enhancement in the distal vessel [5]. Importantly, the temporal definition of >3 months used to define a CTO should be applied based on prior clinical information [6]. Notwithstanding the absence of systematic comparison between CT characteristics of acute versus chronic total coronary occlusions, acute occlusions usually display pronounced positive remodelling and absence of extensive calcifications, whereby the distal vessel segment may lack clear contrast opacification via the collateral circulation [7]. In addition, the non-invasive differentiation between high-grade stenoses and complete occlusions may be challenging due to the limited spatial resolution of current CT scanners, with lesion length ≥9 mm, contrast density difference ≥43% (reflecting intraluminal contrast kinetics over the lesion) and the presence of a ‘reverse attenuation gradient sign’ increasing the likelihood of CTO [8–10]. Notably, non-invasive CTA not only can detect CTO but also adds clues to the natural history of CTO by identification of coronary lesions that later progressed to CTO. Specifically, minimal lumen diameter of <2.0 mm, reference segment diameter of <3.2 mm, and mean plaque attenuation of <50 Hounsfield units on CTA were reported as independent predictors of future CTO lesions [11, 12].

## Advantages of CTO characterisation on CTA

In contrast to invasive angiography, coronary CTA enables complete visualisation of the occlusion site and distal vessel segment [3, 5, 13]. In particular, that coronary CTA can reliably quantify morphological and anatomical details of CTO characteristics (i.e. proximal cap, calcification, tortuosity, occlusion length, and number of occlusion sites) has been repeatedly demonstrated in several trials [2, 14–25]. In addition, the unconstrained number of three-dimensional CT reconstructions facilitates the exact visualisation of the CTO trajectory and aids in selecting the most optimal angiographic projection for orthogonal visualisation of the occluded segment [5, 13]. In general, the more demanding CTO visualisation is on invasive angiography (e.g. patients after coronary artery bypass grafting, ostial occlusions), and/or the more difficult the CTO lesion is to treat (high complexity score, prior failed attempt), the higher is the potential benefit of coronary CTA for planning percutaneous coronary intervention (PCI).

## Coronary CTA for preprocedural planning of CTO PCI

### Prediction of procedural outcome of CTO PCI by coronary CTA

Coronary CTA has been consistently applied for the prediction of time efficiency (i.e. guidewire crossing within 30 min) and the procedural outcome of CTO PCI (i.e. guidewire crossing with restoration of thrombolysis in myocardial infarction grade 3 flow). To this end, several single CTO features as well as combined scoring systems based on CTA have been used to assess the difficulty level of the CTO prior to PCI. Notably, all of the trials on the role of CTA for the prediction of the procedural outcome of CTO PCI were limited by their observational design, and mainly focused on antegrade wiring strategy [2, 14–25].

### Proximal cap

The shape of the proximal cap on angiography is paramount for planning antegrade wiring—whereas a tapered stump indicates the presence of ‘microchannels’ that are usually negotiated with soft polymer-jacketed wires, a blunt stump necessitates the use of stiffer and more penetrating wires [26]. Although coronary CTA can differentiate between a blunt and tapered proximal cap (including the exact origin and direction of the side branches), CT studies have provided conflicting results concerning the influence of stump morphology on success rates in CTO PCI [2, 14, 16, 17, 21, 25]. These disparities may result from varying levels of operator experience and varying CTO PCI techniques across different clinical sites as well as a potentially high variability in the assessment of stump morphology on coronary CTA [25].

**Fig. 1:**
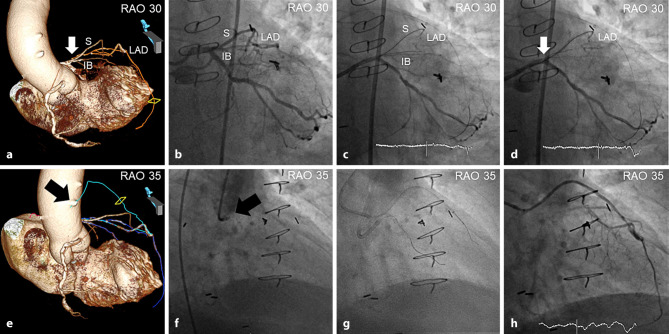
**a**–**h** Coronary computed tomography angiography (*CTA*) for clarification of proximal cap ambiguity and guiding recanalisation of the occluded venous graft for retrograde access to a chronic total occlusion. Three-dimensional CTA volume-rendered reconstruction showing the exact location of the proximal cap of a chronically occluded left anterior descending coronary artery (*LAD*) arising proximal to and above the septal (*S*) branch in the right anterior oblique (*RAO*) view (*arrow*). The exact course of the ostial LAD occlusion traversing between the septal and intermediate branch (*IB*) is displayed (**a**). Invasive coronary angiography failing to show the origin of the chronically occluded LAD (**b**). Advancement of the PILOT 200 guidewire (Abbott Vascular, Santa Clara, CA, USA) into the false lumen along the IB (**c**). Based on CTA images, the PILOT 200 guidewire was retracted and redirected pointing upwards and proximal to the septal branch, tracking the course of the LAD between the septal and IB (**d**). Three-dimensional CTA volume-rendered reconstruction depicting the exact location of the short stump (*black arrow*) as well as the course of the occluded venous graft to the chronically occluded LAD (**e**). Invasive angiography showing the stump (*black arrow*) of the occluded graft to the LAD (**f**). Based on CTA reconstructions aligned with the angulation of the C‑arm, the occluded venous graft was successfully recanalised using the PILOT 200 guidewire supported by a Caravel (Asahi Intecc, Nagoya, Japan) microcatheter (**g,** **h**)

**Fig. 2:**
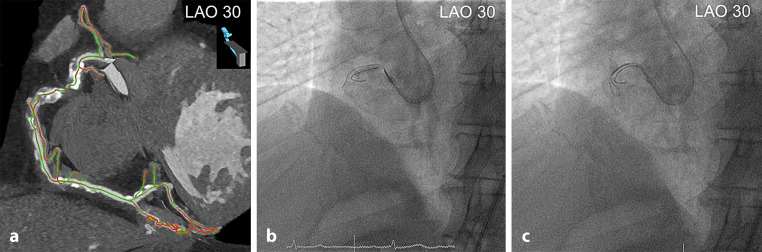
**a**–**c** Coronary computed tomography angiography (*CTA*) for guiding balloon-assisted subintimal entry. Colour-coded CTA multiplanar reconstruction showing the exact course of severely calcified and tortuous chronic total occlusion of the right coronary artery (*RCA*) with adequate length of the proximal segment of the RCA for balloon-assisted subintimal entry. The coronary segments are displayed in *green* (no foreshortening) or *red* (substantial foreshortening) (**a**). Based on CTA reconstructions aligned with the angulation of the C‑arm, a 3.0 × 15 mm balloon was inflated in the proximal RCA, and a Fielder XT‑A guidewire (Asahi Intecc, Nagoya, Japan) was advanced into the subintimal space (**b**). After balloon deflation, the Turnpike Spiral microcatheter (Teleflex, Wayne, PA, USA) was advanced, and a knuckled Fielder XT‑A guidewire was pushed into mid-RCA (**c**). *LAO* left anterior oblique

Apart from assessing the shape of the CTO entry, coronary CTA is uniquely suited for clear visualisation of the proximal cap relative to the further course of the occlusion site—a phenomenon not readily available by any other imaging modality. This virtually precludes the occurrence of an ambiguous cap on CTA, and can be potentially applied for proximal cap clarification in lesions with inconclusive angiographic findings—including guiding of proximal cap puncture (Fig. 1a–d), balloon-assisted subintimal entry (Fig. 2), and the scratch and go technique. In this context, coronary CTA can be also used for localisation of the stumps of the occluded bypass grafts and guiding their recanalisation for retrograde access to CTO PCI (Fig. 1e–h).

### Calcification

The extent and severity of calcifications are proportional to the age of coronary occlusions, and increase the difficulty of CTO PCI [6]. Noteworthy is that CTA is more sensitive in identifying, localising and quantifying calcification as compared with invasive angiography [15, 25]. Several CT studies have consistently demonstrated the independent relationship between calcification and procedural failure in CTO PCI [2, 14–19, 21, 24, 25, 27–29]. Specifically, calcification involving ≥50% of the vessel cross-sectional area (CSA) within the occlusion site (rather than calcification length) is considered the most frequent quantitative correlate of failed CTO PCI [2, 14, 15, 17, 21, 25, 27]. In addition, it was suggested that calcification at the proximal cap (but not at the distal cap) constitutes the major hurdle of successful guidewire crossing when using the antegrade approach [2, 21].

**Fig. 3:**
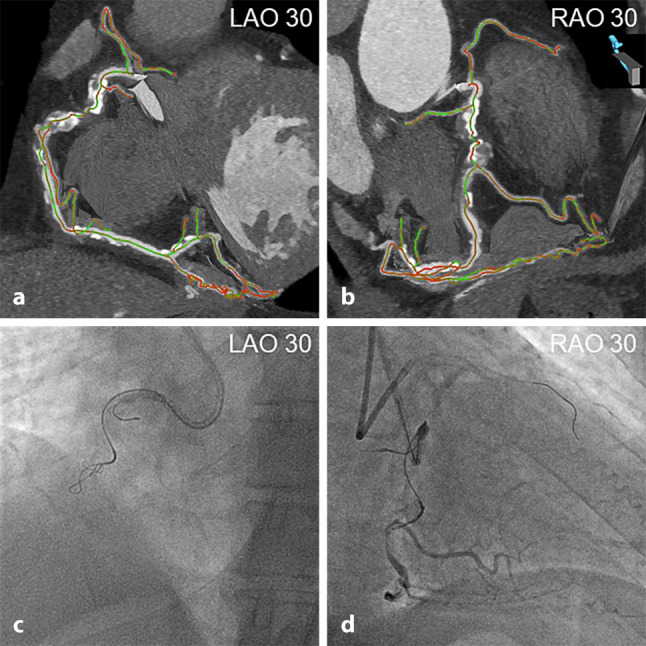
**a**–**d** Coronary computed tomography angiography (*CTA*) for guiding the knuckle wire technique. Colour-coded CTA multiplanar reconstructions demonstrating the exact course of a severely calcified and tortuous CTO of the right coronary artery (*RCA*) with clear delineation of the uptake of the right ventricular branch in right anterior oblique (*RAO*) 30° projection. The coronary segments are displayed in *green* (no foreshortening) or *red* (substantial foreshortening) (**a**, **b**). Based on CTA reconstructions aligned with the angulation of the C‑arm, the knuckled Fielder XT‑A guidewire (Asahi Intecc, Nagoya, Japan) supported by the Turnpike Spiral microcatheter (Teleflex, Wayne, PA, USA) was intentionally advanced along the course of the RCA, and not into the right ventricular branch (**c**, **d**). *LAO* left anterior oblique

Hence, the identification of the extent and severity of calcification (preferably by using the ≥50% cut-off value for vessel CSA) on coronary CTA is paramount for guidewire and microcatheter selection, planning wiring escalation and de-escalation strategies, guiding the knuckle wire technique (Fig. 3), and marking the most optimal re-entry sites in the antegrade dissection and re-entry strategy.

### Tortuosity

CTO tortuosity has significant implications for appropriate guidewire selection. In CTO lesions with marked tortuosity (particularly over long distances), polymer-jacketed wires are preferred due to the lower risk for coronary perforation, whereas straight and short CTO segments can usually be traversed with stiffer wires [26]. Significantly, CTA enables straightforward recognition and precise quantification of coronary tortuosity, which is frequently underestimated on invasive angiography [5, 13]. Particularly germane to this concept, marked vessel tortuosity throughout the occluded segment (most commonly defined as an angle ≥45°) on CTA was repeatedly associated with procedural failure (although not always as an independent predictive factor) [16, 25, 27].

The recognition of marked CTO vessel tortuosity on non-invasive CTA can thus enhance appropriate guidewire selection, and lower the threshold for the dissection and re-entry strategy. In addition, the timely identification of pronounced CTO bending should prompt the operator to choose the optimal fluoroscopic angulation with the least foreshortening of the occlusion site based on three-dimensional CTA reconstructions (Fig. 3).

### Occlusion length and multiple occlusion sites

Angiographic occlusion length ≥ 20 mm has been accepted as a handy and approximate correlate of failed antegrade wiring [28]. Although coronary CTA surpasses invasive angiography in accurate measurement of the occlusion length, the relevance of CT-defined occlusion length in CTO PCI is uncertain [5, 13]. Indeed, while some CT studies indicated the independent association between long occlusions (defined as either >15 mm or >18 mm or ≥32 mm or as a continuous variable) and failed PCI [14, 18, 20, 23, 24, 27], others failed to confirm any independent relationship between occlusion length and CTO PCI outcome [2, 15–17, 19, 21, 22, 25]. These disparities may reflect differences in the application of the hybrid CTO PCI techniques (mainly dissection and re-entry techniques) by different operators for resolving long occlusions.

Besides occlusion length, the relevance of multiple occlusion sites in significantly reducing the chances of time-efficient guidewire crossing has been reported [25]. Specifically, the detrimental effect of multiple occlusions on recanalisation attempts relies on longer occlusion length and multiple entry and exit sites, hampering successful guidewire passage into the distal true lumen. It is noteworthy that coronary CTA is superior to invasive angiography for visualisation of multiple occlusion sites [25].

### CTA-derived scores for prediction of CTO PCI

To date three CTA scoring systems have been developed to predict the procedural outcome of CTO PCI, and thus to grade the CTO difficulty level prior to PCI (Tab. 1). Notably, the trials on CTA-derived scoring systems comprised European, Japanese, and Chinese populations enrolled between 2007 and 2016, and predominantly focused on antegrade wiring strategies [25, 27, 29, 30]. Hence, the clinical applicability of CT scores for prediction of CTO PCI success rates using retrograde wiring and dissection and re-entry strategies is largely unknown.

**Table 1:** Studies on computed tomography angiography-derived scores for prediction of chronic total occlusion (*CTO*) percutaneous coronary intervention

References	Score name	Design	Recruitment period	Type of CT	No. of CTOs	Retrograde approach	External validation
Opolski et al. [25]	CT-RECTOR	Multicentre, retrospective	2007–2013	64/128-slice dual source	240	11%	Yes [27, 31]
Li et al. [29]	J‑CTO_CT_	NR, retrospective	2011–2014	64-slice dual source	171	NR	Yes [30]
Fujino et al. [30]	J‑CTO_CT_	Single-centre, retrospective	2012–2016	320-slice	218	33%	Yes [29]
Yu et al. [27]	KCCT	Multicentre, retrospective	2007–2015	64-slice (including dual source)	456	12%	No

*CT* computed tomography, *NR* not reported

**Fig. 4:**
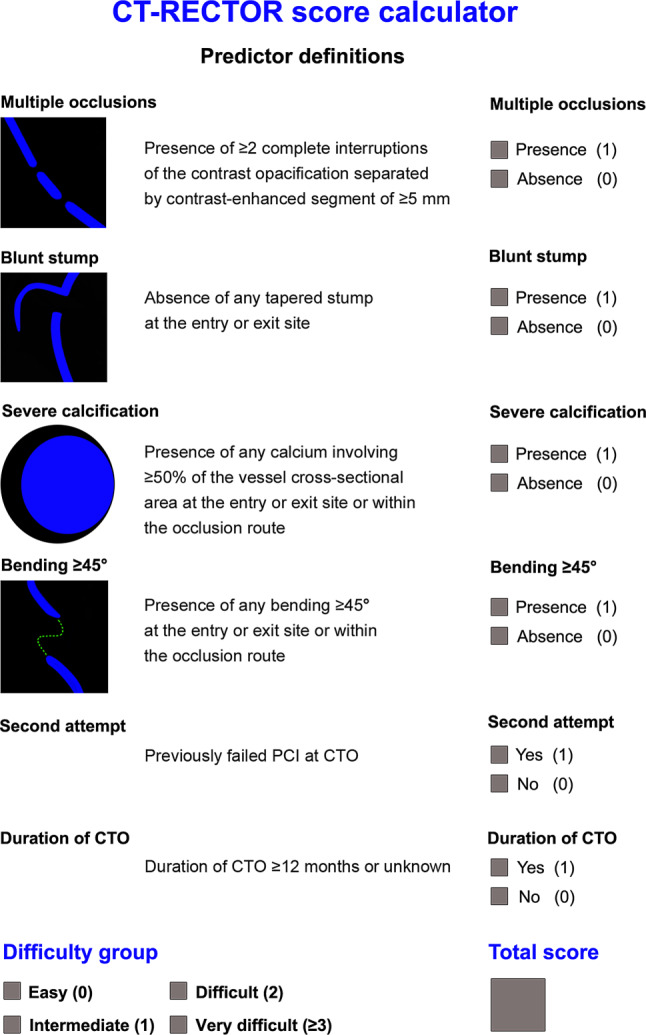
Calculation sheet for the CT-RECTOR (Computed Tomography Registry of Chronic Total Occlusion Revascularization) score with definitions of each variable and listing of the difficulty groups (reprinted with permission from [25]). *CTO* chronic total occlusion, *PCI* percutaneous coronary intervention

Originally, the CT-RECTOR (Computed Tomography Registry of Chronic Total Occlusion Revascularization) score was introduced as an easy-to-use prediction rule for 30-min guidewire crossing derived from 240 CTO lesions from a multicentre European registry [25]. The score features six independent predictors (multiple occlusions, blunt stump, severe calcification occupying ≥50% vessel CSA, bending, CTO age ≥12 months or unknown, previously failed CTO PCI), and is calculated by assigning 1 point for each of the variables and summing all points accrued (Fig. 4). Notably, the CT-RECTOR score was externally validated in a Chinese population as a more accurate prediction rule for 30-min guidewire crossing and final procedural success rate as compared with the angiographic J‑CTO (Multicenter Chronic Total Occlusion Registry of Japan) score [31]. Subsequently, two trials explored the predictive ability of the J‑CTO score derived from CTA (the so-called J‑CTO_CT_ score) [29, 30]. Interestingly, while in one study the non-invasive J‑CTO score determined by CTA yielded a similar predictive value for 30-min guidewire crossing as compared with the angiographic J‑CTO score [29], the other study demonstrated significantly higher discriminating accuracy of the J‑CTO_CT_ score than that of the angiography-based J‑CTO score [30]. Noteworthy is that the J‑CTO scores determined by coronary CTA and invasive angiography differ in the amount of surveyed calcification—while on CTA calcification must involve >50% of the vessel CSA, invasive angiography stratifies the presence or absence of calcification only. Finally, the KCCT (Korean Multicenter CTO CT Registry) score was developed in the derivation cohort of 456 CTO lesions, yielding seven independent predictors (proximal blunt entry, proximal side branch, bending, occlusion length ≥15 mm, severe calcification or whole luminal calcification, reattempt, and ≥12 months or unknown duration of occlusion) of 30-min guidewire crossing [27]. Whereas it showed higher discriminative performance compared with the other scoring systems, including J‑CTO, CL (clinical and lesion-related), PROGRESS-CTO (Prospective Global Registry for the Study of Chronic Total Occlusion Intervention), and CT-RECTOR scores (with the least numerical difference between KCCT and CT-RECTOR score), some of the parameters included in the KCCT score (e.g. proximal side branch, occlusion length ≥15 mm) may be less relevant when hybrid techniques (e.g. balloon-assisted subintimal entry) and modern CTO PCI devices are applied [26, 32].

In summary, clinicians may find the CTA-based scores particularly useful to better estimate the time and resources required for the interventional treatment of CTO, and to plan training sessions for less-skilled interventionalists.

## Coronary CTA for periprocedural guidance of CTO PCI

### Coronary CTA co-registration in the catheterisation laboratory

Apparently, the biggest advantage of coronary CTA for facilitating CTO PCI relies on real-time CTA co-registration in the catheterisation laboratory [13]. To this end, one approach has been to import preprocedural CT datasets directly to the angiography system with subsequent display of CTA images on separate monitors. Specifically, such a solution features a fully automatic alignment of CT reconstructions according to the angulation of the C‑arm, enabling a continuous three-dimensional CTA roadmap during PCI [5, 13]. In addition, the colour-coded display of coronary arteries in virtual CTA facilitates contrast-free identification of fluoroscopic projections without foreshortening for each respective vessel segment [5]. This has been corroborated in a study by Rolf et al., who reported a significantly higher CTO recanalisation success rate in 25 patients with CTA co-registration as compared with 25 patients without CTA overlay [33]. Subsequently, the periprocedural co-registration of CTA using a wearable computer resulted in improved efficiency and less use of resources (as reflected by more frequent selection of the first-choice stiffer wire and lower contrast exposure), although there were no differences in CTO success rate as compared with standard CTO PCI [34]. Notably, both of these studies were limited by observational design and antegrade wiring strategies, so that the results need to be interpreted with caution [33, 34].

### Coronary CTA/fluoroscopy fusion guidance

Recently, a more challenging approach involving real-time fusion of three-dimensional coronary CTA with X‑ray fluoroscopy for guiding CTO PCI has been investigated. In a study by Ghoshhajra et al. among 24 consecutive CTO PCI patients, CTA/fluoroscopy fusion provided new insights into the extent and localisation of coronary calcification as well as vessel tortuosity, potentially influencing antegrade wiring, antegrade and dissection re-entry, and retrograde wiring strategies [35]. Similar findings were reported by Xenogiannis et al., in whose study CTA/fluoroscopy fusion was used in 27 of 146 CTO PCI cases performed between 2018 and 2019 within the PROGRESS-CTO registry [36]. Noteworthy, the most common indication for CTA/fluoroscopy fusion was to clarify proximal cap ambiguity, followed by facilitation of intra-CTO wire advancement and re-entry. This subsequently contributed to antegrade and dissection re-entry being the most common successful crossing technique in the CTA/fluoroscopy fusion group—a phenomenon with a potential for reducing the need for a retrograde approach. Notably, although both of these studies were small and had an observational design, they are the first to examine the influence of coronary CTA for guiding CTO PCI using the hybrid algorithm [35, 36].

### Periprocedural CTA scanning in the catheterisation laboratory

Finally, the interchangeable CTA scanning and analysis intertwined with invasive fluoroscopy has been tested in a series of 61 patients undergoing CTO PCI to investigate the role of intraprocedural CTA for identification of guidewire tip locations [37]. Whereas on-site acquisition and review of the CT images in the catheterisation laboratory was safe and feasible, not every CTA result provided precise information about guidewire location (not to mention potentially higher labour input and radiation dose of intraprocedural CTA).

## Considerations for implementation of coronary CTA within the hybrid algorithm

**Fig. 5:**
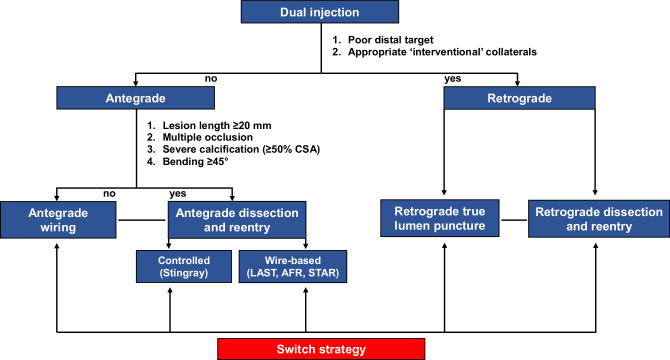
Computed tomography angiography (*CTA*)-based hybrid algorithm for crossing a chronic total occlusion (*CTO*). The algorithm is based on a meticulous review of coronary CTA and dual-injection invasive angiography. Based on the premise that coronary CTA enables clear visualisation of the CTO entry relative to the further course of the occlusion site, the proximal cap does not determine the choice of either an antegrade or a retrograde strategy. Thus, only two CTO features determine whether the primary strategy is antegrade or retrograde: (1) the quality of the distal vessel; and (2) interventional collaterals. Also, occlusion length alone does not determine the choice of either a wire escalation strategy or a dissection and re-entry strategy, but rather a combination of factors including multiple occlusion sites, severe calcification (≥50% vessel cross-sectional area (*CSA*)), bending (≥45°), and occlusion length ≥20 mm. Swift changes of strategies are encouraged in case of failure in order to boost procedural efficiency. *AFR* antegrade fenestration and re-entry, *LAST* limited antegrade subintimal tracking, *STAR* subintimal tracking and re-entry

The hybrid algorithm was developed by North American operators in 2012 and is based on the concept of a rapid change of one strategy to another in case of failure, in order to boost procedural efficiency [38]. Herein, we propose a modified hybrid algorithm for CTO PCI based on coronary CTA—namely a CTA-based hybrid algorithm (Fig. 5). To this end, the availability of coronary CTA co-registration aligned with the angulation of the C‑arm during CTO PCI is highly recommended.

**Fig. 6:**
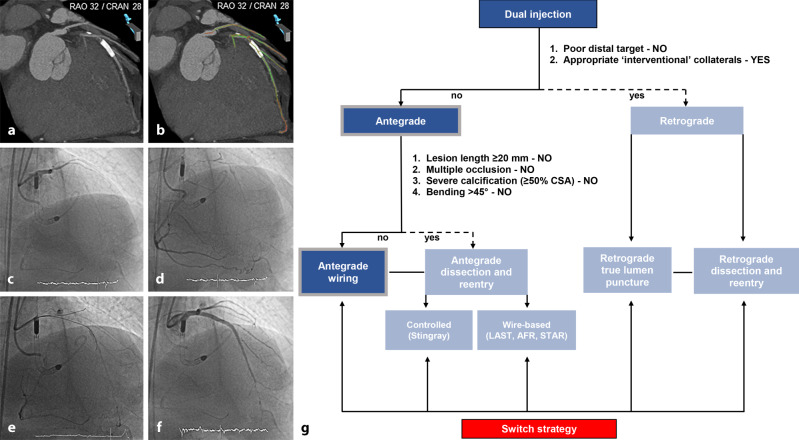
**a**–**g** Antegrade wire escalation strategy of a chronically occluded left anterior descending coronary artery (*LAD*). Coronary computed tomography angiography (*CTA*) multiplanar reconstruction revealing a tapered proximal cap, a short non-calcified occlusion site without bending and a good distal target (**a**). Colour-coded CTA multiplanar reconstruction displayed in the catheterisation laboratory for guidance of antegrade wiring. The coronary segments are displayed in *green* (no foreshortening) or *red* (substantial foreshortening) (**b**). Baseline dual invasive angiography demonstrating a chronic total occlusion (*CTO*) of the ostial LAD with distal filling via collaterals from the right coronary artery (**c**, **d**). Based on the CTA-based hybrid algorithm, antegrade wiring was chosen as the primary recanalisation strategy, and the CTO was successfully crossed with a Fielder XT‑A guidewire (Asahi Intecc, Nagoya, Japan) (**e**). Final angiographic result after the implantation of multiple drug-eluting stents with restoration of antegrade thrombolysis in myocardial infarction grade 3 flow (**f**). The CTA-based hybrid algorithm for the presented case (**g**). *AFR* antegrade fenestration and re-entry, *CRAN* cranial, *CSA* cross-sectional area, *LAST* limited antegrade subintimal tracking, *RAO* right anterior oblique, *STAR* subintimal tracking and re-entry

**Fig. 7:**
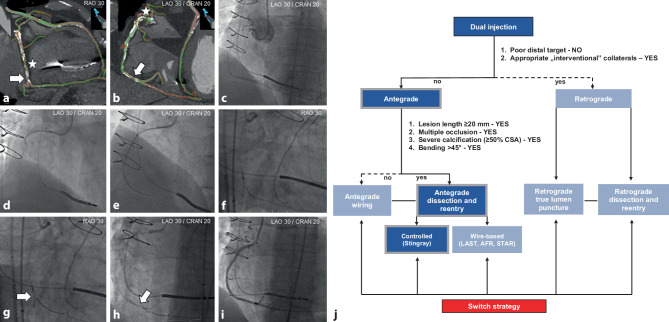
**a**–**i** Antegrade dissection and re-entry strategy of a chronically occluded right coronary artery (*RCA*). Coronary computed tomography angiography (*CTA*) multiplanar reconstructions revealing short and tapered proximal cap, long and severely calcified double occlusion site (*stars*) with bending and a relatively good distal target with multiple calcium spots. The *white arrows* indicate the favourable re-entry sites within the distal RCA (**a**, **b**). Baseline dual invasive angiography demonstrating a complex chronic total occlusion of the RCA with distal filling via collaterals from the left anterior descending coronary artery supplied by the left internal mammary artery (**c**). Based on the CTA-based hybrid algorithm, an antegrade dissection and re-entry strategy was selected as the primary recanalisation approach, and a knuckle Fielder XT‑A guidewire (Asahi Intecc, Nagoya, Japan) was advanced to the mid-RCA (**d**). After antegrade insertion of a GuideLiner (Teleflex, Wayne, PA, USA), the CrossBoss catheter (Boston Scientific, Boston, MA, USA) crossed the occlusion subintimally into the distal RCA (**e**). Subsequently, the CrossBoss catheter was exchanged for a Stingray balloon (Boston Scientific, Boston, MA, USA), placed adjacent to the true lumen in the distal RCA between calcium spots as indicated on CTA images (**f**). After multiple sticks with a Confianza Pro 12 guidewire (Asahi Intecc, Nagoya, Japan), the Gladius guidewire (Asahi Intecc, Nagoya, Japan) successfully crossed into the distal true lumen (*arrows*, **g**, **h**). Final angiographic result after implantation of multiple drug-eluting stents with restoration of antegrade thrombolysis in myocardial infarction grade 3 flow (**i**). The CTA-based hybrid algorithm for the presented case (**j**). *AFR* antegrade fenestration and re-entry, *CRAN* cranial, *LAO* left anterior oblique, *LAST* limited antegrade subintimal tracking, *RAO* right anterior oblique, *STAR* subintimal tracking and re-entry

In particular, all CTO features are simultaneously evaluated based on invasive angiography and coronary CTA. In contrast to the original hybrid algorithm, the proximal cap does not determine the choice of either an antegrade or a retrograde approach based on the premise that CTO lesions have a clear (non-ambiguous) proximal cap on coronary CTA. Notably, two remaining angiographic parameters (namely the quality and size of the distal vessel and ‘interventional collaterals’) dictating whether the primary approach is antegrade or retrograde are retained. In addition, in contrast to the original hybrid approach, occlusion length alone does not dictate the choice of either an antegrade wire escalation strategy or an antegrade dissection and re-entry strategy, but rather a combination of factors including multiple occlusion sites, severe calcification (≥50% vessel CSA), bending (≥45°), and occlusion length (Figs. 6 and 7). This is based on numerous CT studies that have identified the above-mentioned factors as significant predictors for failed guidewire crossing [2, 14–25, 27, 29–31]. Importantly, the threshold for antegrade dissection and re-entry as an initial CTO PCI strategy is lowered based on the clear delineation of both the proximal cap and the distal CTO segment (including the extent and severity of calcifications) on coronary CTA, thereby potentially reducing the need for the retrograde approach. In contrast, complex lesions with poor distal targets (including bifurcation at distal cap) and good interventional collaterals favour an initial retrograde approach.

## Conclusion

The ability of CTA to identify and quantify coronary atherosclerotic plaque, as well as to project three-dimensional coronary vessel reconstructions, has garnered particular attention in the context of preprocedural planning and periprocedural guidance of CTO PCI. To this end, several CTO features on coronary CTA (e.g. severe calcification, bending, multiple occlusion sites) have been used to predict the procedural success rate of CTO PCI. More importantly, combined CTA scoring systems (e.g. CT-RECTOR score) have been developed to gauge the time required for successful guidewire crossing, and thus to grade the level of difficulty of the CTO prior to PCI. Significantly, the CTA-based scores have been validated as more accurate prediction tools compared with the angiographic J‑CTO score, lending support to improved treatment and training strategies as well as allocation of resources in the catheterisation laboratory. Moreover, the introduction of automated software tools enabling real-time CTA/fluoroscopy co-registration can aid in periprocedural guidance of CTO PCI based on three-dimensional CTA information. Specifically, it offers the unprecedented opportunity to resolve proximal cap ambiguity and clearly visualise the distal CTO segment (not to mention accurate identification of severe calcification, multiple occlusion sites, and bending), thereby potentially influencing CTO PCI strategies and techniques. In this regard, we have introduced a CTA-based hybrid algorithm with potential for further enhancing the efficiency of CTO PCI. Importantly, this coincides with wider adoption and growing indications for coronary CTA in the diagnostic work-up of patients with chronic coronary syndromes. Further studies are needed to confirm the time efficiency and procedural success rates of CTO PCI using a CTA-based hybrid algorithm.

## References

[1] Opolski MP, Hartaigh BÓ, Berman DS, Budoff MJ, Achenbach S, Al-Mallah M (2015). Current trends in patients with chronic total occlusions undergoing coronary CT angiography. Heart.

[2] García-García HM, van Mieghem CA, Gonzalo N, Meijboom WB, Weustink AC, Onuma Y (2009). Computed tomography in total coronary occlusions (CTTO registry): radiation exposure and predictors of successful percutaneous intervention. EuroIntervention.

[3] Opolski MP, Kepka C, Achenbach S, Juraszynski Z, Pregowski J, Kruk M (2012). Coronary computed tomographic angiography for prediction of procedural and intermediate outcome of bypass grafting to left anterior descending artery occlusion with failed visualization on conventional angiography. Am J Cardiol.

[4] Opolski MP, Gransar H, Lu Y, Achenbach S, Al-Mallah MH, Andreini D (2019). Prognostic value of chronic total occlusions detected on coronary computed tomographic angiography. Heart.

[5] Opolski MP, Achenbach S (2015). CT Angiography for revascularization of CTO: crossing the borders of diagnosis and treatment. JACC Cardiovasc Imaging.

[6] Stone GW, Kandzari DE, Mehran R, Colombo A, Schwartz RS, Bailey S (2005). Percutaneous recanalization of chronically occluded coronary arteries: a consensus document: part I. Circulation.

[7] Lehman SJ, Schlett CL, Bamberg F, Nieman K, Abbara S, Hoffmann U (2008). Appearance of acute and chronic coronary occlusions in contrast-enhanced cardiac computed tomography. JACC Cardiovasc Imaging.

[8] von Erffa J, Ropers D, Pflederer T, Schmid M, Marwan M, Daniel WG (2008). Differentiation of total occlusion and high-grade stenosis in coronary CT angiography. Eur Radiol.

[9] Staruch AD, Opolski MP, Slomka PJ, Staruch M, Kepka C, Witkowski A (2016). Automated quantitative plaque analysis for discrimination of coronary chronic total occlusion and subtotal occlusion in computed tomography angiography. J Thorac Imaging.

[10] Li M, Zhang J, Pan J, Lu Z (2013). Obstructive coronary artery disease: reverse attenuation gradient sign at CT indicates distal retrograde flow—a useful sign for differentiating chronic total occlusion from subtotal occlusion. Radiology.

[11] Kang J, Chun EJ, Park HJ, Cho YS, Park JJ, Kang SH (2019). Clinical and computed tomography angiographic predictors of coronary lesions that later progressed to chronic total occlusion. JACC Cardiovasc Imaging.

[12] Opolski MP (2019). Noninvasive precursors of coronary chronic total occlusions: fantasy or reality?. JACC Cardiovasc Imaging.

[13] Opolski MP (2018). Cardiac computed tomography for planning revascularization procedures. J Thorac Imaging.

[14] Mollet NR, Hoye A, Lemos PA, Cademartiri F, Sianos G, McFadden EP (2005). Value of preprocedure multislice computed tomographic coronary angiography to predict the outcome of percutaneous recanalization of chronic total occlusions. Am J Cardiol.

[15] Soon KH, Cox N, Wong A, Chaitowitz I, Macgregor L, Santos PT (2007). CT coronary angiography predicts the outcome of percutaneous coronary intervention of chronic total occlusion. J Interv Cardiol.

[16] Ehara M, Terashima M, Kawai M, Matsushita S, Tsuchikane E, Kinoshita Y (2009). Impact of multislice computed tomography to estimate difficulty in wire crossing in percutaneous coronary intervention for chronic total occlusion. J Invasive Cardiol.

[17] Cho JR, Kim YJ, Ahn C-M, Moon J-Y, Kim J-S, Kim H-S (2010). Quantification of regional calcium burden in chronic total occlusion by 64-slice multidetector computed tomography and procedural outcomes of percutaneous coronary intervention. Int J Cardiol.

[18] Li P, Gai L, Yang X, Sun ZJ, Jin QH (2010). Computed tomography angiography-guided percutaneous coronary intervention in chronic total occlusion. J Zhejiang Univ Sci B.

[19] Hsu JT, Kyo E, Chu CM, Tsuji T, Watanabe S (2011). Impact of calcification length ratio on the intervention for chronic total occlusions. Int J Cardiol.

[20] Choi J-H, Song YB, Hahn J-Y, Choi SH, Gwon H-C, Cho JR (2011). Three-dimensional quantitative volumetry of chronic total occlusion plaque using coronary multidetector computed tomography. Circ J.

[21] Martín-Yuste V, Barros A, Leta R, Ferreira I, Brugaletta S, Pujadas S (2012). Factors determining success in percutaneous revascularization of chronic total coronary occlusion: multidetector computed tomography analysis. Rev Esp Cardiol.

[22] Li M, Zhang J, Pan J, Lu Z (2013). Coronary total occlusion lesions: linear intrathrombus enhancement at CT predicts better outcome of percutaneous coronary intervention. Radiology.

[23] Luo C, Huang M, Li J, Liang C, Zhang Q, Liu H (2015). Predictors of interventional success of antegrade PCI for CTO. JACC Cardiovasc Imaging.

[24] Chen Y, Lu B, Hou Z, Gao Y, Yu F-F, Yin W-H (2015). Predicting successful percutaneous coronary intervention in patients with chronic total occlusion: the incremental value of a novel morphological parameter assessed by computed tomography. Int J Cardiovasc Imaging.

[25] Opolski MP, Achenbach S, Schuhbäck A, Rolf A, Möllmann H, Nef H (2015). Coronary computed tomographic prediction rule for time-efficient guidewire crossing through chronic total occlusion: insights from the CT-RECTOR multicenter registry (computed tomography registry of chronic total occlusion revascularization). JACC Cardiovasc Interv.

[26] Brilakis ES, Mashayekhi K, Tsuchikane E, Rafeh NA, Alaswad K, Araya M (2019). Guiding principles for chronic total occlusion percutaneous coronary intervention. Circulation.

[27] Yu CW, Lee HJ, Suh J, Lee NH, Park SM, Park TK (2017). Coronary computed tomography angiography predicts guidewire crossing and success of percutaneous intervention for chronic total occlusion: Korean multicenter CTO CT registry score as a tool for assessing difficulty in chronic total occlusion percutaneous coronary intervention. Circ Cardiovasc Imaging.

[28] Morino Y, Abe M, Morimoto T, Kimura T, Hayashi Y, Muramatsu T (2011). Predicting successful guidewire crossing through chronic total occlusion of native coronary lesions within 30 min: the J-CTO (multicenter CTO registry in Japan) score as a difficulty grading and time assessment tool. JACC Cardiovasc Interv.

[29] Li Y, Xu N, Zhang J, Li M, Lu Z, Wie M (2015). Procedural success of CTO recanalization: comparison of the J-CTO score determined by coronary CT angiography to invasive angiography. J Cardiovasc Comput Tomogr.

[30] Fujino A, Otsuji S, Hasegawa K, Arita T, Takiuchi S, Fujii K (2018). Accuracy of J-CTO score derived from computed tomography versus angiography to predict successful percutaneous coronary intervention. JACC Cardiovasc Imaging.

[31] Tan Y, Zhou J, Zhang W, Zhou Y, Du L, Tian F (2017). Comparison of CT-RECTOR and J-CTO scores to predict chronic total occlusion difficulty for percutaneous coronary intervention. Int J Cardiol.

[32] Schumacher SP, Stuijfzand WJ, Opolski MP, van Rossum AC, Nap A, Knaapen P (2019). Percutaneous coronary intervention of chronic total occlusions: when and how to treat. Cardiovasc Revasc Med.

[33] Rolf A, Werner GS, Schuhbäck A, Rixe J, Möllmann H, Nef HM (2013). Preprocedural coronary CT angiography significantly improves success rates of PCI for chronic total occlusion. Int J Cardiovasc Imaging.

[34] Opolski MP, Debski A, Borucki BA, Staruch AD, Kepka C, Rokicki JK (2017). Feasibility and safety of augmented-reality glass for computed tomography-assisted percutaneous revascularization of coronary chronic total occlusion: a single center prospective pilot study. J Cardiovasc Comput Tomogr.

[35] Ghoshhajra BB, Takx RA, Stone LL, Girard EE, Brilakis ES, Lombardi WL (2017). Real-time fusion of coronary CT angiography with x-ray fluoroscopy during chronic total occlusion PCI. Eur Radiol.

[36] Xenogiannis I, Jaffer F, Shah AR, Omer M, Megaly M, Vemmou E (2020). Computed tomography angiography co-registration with real-time fluoroscopy in chronic total occlusion percutaneous coronary interventions. EuroIntervention.

[37] Kim BK, Cho I, Hong MK, Chang HJ, Shin DH, Kim JS (2016). Usefulness of intraprocedural coronary computed tomographic angiography during intervention for chronic total coronary occlusion. Am J Cardiol.

[38] Brilakis ES, Grantham JA, Rinfret S, Wyman RM, Burke MN, Karmpaliotis D (2012). A percutaneous treatment algorithm for crossing coronary chronic total occlusions. JACC Cardiovasc Interv.

